# Risk factors for bacteremic pneumonia and mortality (28-day mortality) in patients with *Acinetobacter baumannii* bacteremia

**DOI:** 10.1186/s12879-024-09335-8

**Published:** 2024-04-26

**Authors:** Meng Lan, Kang Dongmei, Shen Guodong, Yao Haifeng, Cui Guofeng, Chen Mengting, Fan Xiaoyun

**Affiliations:** 1 Department of Geriatric Respiratory and Critical Care Medicine, The First Affiliated Hospital of Anhui Medical University, Hefei, Anhui 230032 China; 2 International Medicine Depaterment, Division of Life Science and Medicine, The First Affiliated Hospital of USTC, University of Science and Technology of China, Hefei, 230001 Anhui China; 3 Anhui Province Key Laboratory of Geriatric Immunology and Nutrition Therapy, Division of Life Sciences and Medicine, The First Affiliated Hospital of USTC, University of Science and Technology of China, Hefei, 230001 Anhui China; 4 Information Center, Division of Life Sciences and Medicine, The First Affiliated Hospital of USTC, University of Science and Technology of China, Hefei, 230001 Anhui China; 5 Anhui Geriatric Institute, the First Affiliated Hospital of Anhui Medical University, Hefei, Anhui, 230022 PR China

**Keywords:** *Acinetobacter baumannii*, Bacteremia, Pneumonia, Risk factors, Surgery, Survival time

## Abstract

**Background:**

Patients infected with *Acinetobacter baumannii* (AB) bacteremia in hospital have high morbidity and mortality. We analyzed the clinical characteristics of pneumonia and nonpneumonia-related AB bloodstream infections (AB BSIs) and explored the possible independent risk factors for the incidence and prognosis of pneumonia-related AB BSIs.

**Methods:**

A retrospective monocentric observational study was performed. All 117 episodes of hospital-acquired AB bacteremia sorted into groups of pneumonia-related AB BSIs (*n* = 45) and nonpneumonia-related AB BSIs (*n* = 72) were eligible. Univariate/multivariate logistic regression analysis was used to explore the independent risk factors. The primary outcome was the antibiotic susceptibility in vitro of pneumonia-related AB BSIs group. The secondary outcome was the independent risk factor for the pneumonia-related AB BSIs group.

**Results:**

Among 117 patients with AB BSIs, the pneumonia-related group had a greater risk of multidrug resistant *A. baumannii* (MDRAB) infection (84.44%) and carbapenem-resistant *A. baumannii* (CRAB) infection (80%). Polymyxin, minocycline and amikacin had relatively high susceptibility rates (> 80%) in the nonpneumonia-related group. However, in the pneumonia-related group, only polymyxin had a drug susceptibility rate of over 80%. Univariate analysis showed that survival time (day), CRAB, MDRAB, length of hospital stay prior to culture, length of ICU stay prior to culture, immunocompromised status, antibiotics used prior to culture (n > = 3 types), endotracheal tube, fiberoptic bronchoscopy, PITT, SOFA and invasive interventions (n > = 3 types) were associated with pneumonia-related AB bacteremia. The multivariate logistic regression analysis revealed that recent surgery (within 1 mo) [*P* = 0.043; 0.306 (0.098–0.962)] and invasive interventions (n > = 3 types) [*P* = 0.021; 0.072 (0.008–0.671)] were independent risk factors related to pneumonia-related AB bacteremia. Multivariate logistic regression analysis revealed that length of ICU stay prior to culture [*P* = 0.009; 0.959 (0.930–0.990)] and recent surgery (within 1 mo) [*P* = 0.004; 0.260 (0.105–0.646)] were independent risk factors for mortality in patients with pneumonia-related AB bacteremia. The Kaplan‒Meier curve and the timing test showed that patients with pneumonia-related AB bacteremia had shorter survival time compared to those with nonpneumonia-related AB bacteremia.

**Conclusions:**

Our study found that *A. baumannii* had a high rate of antibiotic resistance in vitro in the pneumonia-related bacteremia group, and was only sensitive to polymyxin. Recent surgery was a significantly independent predictor in patients with pneumonia-related AB bacteremia.

**Supplementary Information:**

The online version contains supplementary material available at 10.1186/s12879-024-09335-8.

## Background

*Acinetobacter baumannii* (*A. baumannii*), a ubiquitous gram-negative bacillus, is frequently distributed in intensive care unit (ICU) environments, colonizes human mucosal surfaces and medical devices, and has become one of the most prominent opportunistic nosocomial pathogens [1, 2]. *A. baumannii* infection primarily presents as bloodstream infection (bacteremia), pneumonia infection and abdominal infection [3]. Compared with *A. baumannii* infections at other sites, *A. baumannii* bacteremia acquired more attention due to its higher mortality, longer hospital stays and greater costs [4, 5]. Therefore, *A. baumannii* bacteremia has become a major global health crisis.

Various antibiotic-resistant *A. baumannii* strains have emerged due to the widespread use of antibiotics, especially carbapenem-resistant *A. baumannii* (CRAB) strains and multidrug-resistant *A. baumannii* (MDRAB) strains [6, 7]. In recent years, the global prevalence of CRAB and MDRAB has gradually increased in patients with AB bacteremia [8, 9]. A recent report said that *A. baumannii* bacteremia is the most severe clinical type because the mortality was as high as 58.24% in cases of CRAB bacteremia [10]. The use of antibiotics in treating AB bacteremia caused by CRAB and MDRAB is highly limited. Therefore, bacterial drug resistance monitoring has positive significance for understanding the changes in drug resistance and guiding rational clinical drug use.

Some prognostic factors, such as basic diseases, bacteremia sources, surgery, invasion procedure, mechanical ventilation, decreased immunity, length of stay in the ICU and length of hospital stay, play important roles in predicting clinical outcomes. Objective quantitative assessments of severity are more important in treatment decisions. Several organ dysfunction scoring systems have been developed to assess the prognosis of critically ill patients [11]. The Acute Physiology and Chronic Health Evaluation II (APACHE II) [4], Sequential Organ Failure Assessment (SOFA) [12] and Pitt bacteremia score (Pitt) [13] are the three most commonly used scoring systems for organ dysfunction.

Many studies have shown that the epidemiology of AB bacteremia greatly depends on the region, year, hospital ward and even infection sites. Therefore, it is necessary to investigate changes in the microbiological characteristics, prevalence, treatments and prognosis in patients with AB bacteremia. Yihai Gu et al. revealed that primary infection in the central nervous system is independently associated with bacteremia caused by *A. baumannii*. In addition, a recent study found that patients with pneumonia had a significantly higher incidence of AB bacteremia and antibiotic resistance, longer hospital stays and higher mortality [14]. Zhou et al. [10] and Liu et al. [15] suggested that a respiratory tract bacteremia origin may be an independent risk factor for mortality. However, another study showed that primary infection in the respiratory system was independently associated with a decreased risk of bacteremia [15]. Therefore, we performed this single-center retrospective study to analyse different microbiological and clinical characteristics between patients with pneumonia and nonpneumonia-related AB bacteremia and then explore the independent risk factors for the incidence and prognosis of patients with pneumonia-related AB bacteremia.

## Materials and methods

### Study design and patient enrollment

This retrospective observational cohort study was conducted at the First Affiliated Hospital (Anhui Provincial Hospital) of the University of Science and Technology of China, including four branch areas: the central courtyard area, the southern district, the western district and the infectious disease hospital. Apart from the respiratory tract sources (38.5%), the nonpneumonia-related AB BSIs was sources from primary (22.2%), urinary tract (2.6%), catheter (3.4%), intra-abdominal (20.5%) and skin-soft tissue (12.8%) in the total number of 117patients. Because of the retrospective and observational nature of the study, the institutional review board waived the requirement for informed consent. The study followed the principles of the Declaration of Helsinki and STROBE guidelines.

### Inclusion criteria

We enrolled 117 patients (aged ≥ 18 years) with AB bacteremia admitted between June 1, 2020, and September 30, 2023. If a patient had multiple hospital records during the study period, only the first visit was included.

### Exclusion criteria

The following conditions were excluded: (1) Those who were transferred out or died or gave up treatment within 24 h after admission; (2) Incomplete clinical data; (3) Persons under the age of 18.

### Data collection and study definitions

#### Antimicrobial susceptibilities and definition of drug resistance in *Acinetobacter baumannii*

The VITEK 2 Compact system or MALDI-TOF MS were used to identify AB isolates, and the VITEK-2 Compact AST-GN16 or Kirby-Bauer test were used to determine in vitro antimicrobial susceptibilities. According to the Clinical and Laboratory Standards Institute (CLSI) standards, a minimum inhibitory concentration (MIC) ≥ 8 µg/mL for imipenem and meropenem was considered to indicate carbapenem resistance. Cefoperazone-sulbactam susceptibility was determined based on the breakpoints for ampicillin sulbactam (MIC 16/8 µg/mL). The United States Food and Drug Administration breakpoints were used to determine tigecycline susceptibility. Susceptibility to other antibiotics was determined based on CLSI standards. Carbapenem-resistant *A. baumannii* (CRAB) exhibits antimicrobial resistance to imipenem and meropenem at the same time. Multidrug-resistant (MDR) Acinetobacter refers to the drug resistance of three or more types of antibacterial drugs (mainly cephalosporins and carbapenems against Pseudomonas, compound preparations containing β-lactamase inhibitors, fluoroquinolones, and aminoglycosides) that have potential antibacterial activity against the bacterium. The resistance and susceptibility results of pneumonia and nonpneumonia-related AB bacteremia are listed in Tables 1 and 2, respectively.

**Table 1:** In vitro resistance results of pneumonia and nonpneumonia-related *A. baumannii* bacteremia

Antibiotic types	Total (*n* = 117)	Pneumonia-related bacteremia (*n* = 45)	Nonpneumonia-related bacteremia (*n* = 72)	Χ^2^	*P*
**Levofloxacin** **Ceftazidime** **Imipenem** **Piperacillin/ tazobactam** **Ciprofloxacin** **Minocycline** **Tobramycin** **Meropenem** **Cefepime** **Ticarcillin/ clavulanic** **acid** **Cefopetazone/ sulbactam** **Doxycycline** **Compound Xinnuomin** **Amikacin** **Tigecycline** **Colistin** **CR-AB** **MDR-AB**	**50.4%(59/117)** **60.6%(71/117)** **58.9%(69/117)** **67%(67/100)** **60.75%(65/107)** **16.48%(15/91)** **54.2%(58/107)** **78.89%(62/90)** **55.5%(65/117)** **70%(63/90)** **52.94%(54/102)** **57.14%(52/91)** **48.7%(57/117)** **17.39%(4/23)** **5.88%(6/102)** **3.37%(3/91)** **58.97%(69/117)** **61.53%(72/117)**	**77.7%(35/45)** **82.2%(37/45)** **80%(36/45)** **83.72%(36/43)** **86.05%(37/43)** **26.83%(11/41)** **76.74%(33/43)** **77.7%(35/45)** **75.5%(34/45)** **85.36%(35/41)** **69.76%(30/43)** **73.17%(30/41)** **60%(27/45)** **40%(2/5)** **6.98%(3/43)** **2.44%(1/41)** **80%(36/45)** **84.44%(38/45)**	**33.3%(24/72)** **47.2%(34/72)** **45.8%(33/72)** **54.38%(31/57)** **43.75%(28/64)** **8%(4/50)** **39.07%(25/64)** **56%(28/50)** **43%(31/72)** **57.14%(28/49)** **40.68%(24/59)** **44%(22/50)** **41.6%(30/72)** **11.11%(2/18)** **5.08%(3/59)** **4.17%(2/48)** **45.83%(33/72)** **47.22%(34/72)**	**21.88** **14.21** **8.03** **9.54** **19.29** **5.8** **15.29** **8.72** **11.84** **8.46** **3.44** **7.82** **3.72** **2.27** **0.15** **0.20** **13.36** **16.21**	**0** **0** **0.006** **0.03** **0** **0.023** **0** **0.005** **0.001** **0.004** **0.005** **0.006** **0.06** **0.19** **0.69** **1** **0** **0**

**Table 2:** In vitro susceptibility results of pneumonia and non-pneumonia-related *A. baumannii* bacteremia to major drugs

Antibiotic types	Total (*n* = 117)	Pneumonia-related bacteremia (*n* = 45)	Nonpneumonia- related bacteremia (*n* = 72)	Χ^2^	*P*
**Minocycline** **Amikacin** **Meropenem** **Imipenem** **Tigecycline** **Colistin**	**62.64%(57/91)** **73.91%(17/23)** **31.11%(28/90)** **41%(48/117)** **69.61%(71/102)** **96.63%(86/89)**	**39.02%(16/41)** **20%(1/5)** **15%(6/40)** **20%(9/45)** **58.14%(25/43)** **97.56%(40/41)**	**82%(41/50)** **88.89%(16/18)** **44%(30/72)** **54.1%(39/72)** **77.96%(46/59)** **95.83%(46/48)**	**17.77** **9.63** **8.38** **13.36** **4.62** **0.2**	**0** **0.008** **0.006** **0** **0.049** **1**

### General data

We collected data on demographic characteristics (sex, age), underlying diseases (history of smoking and alcohol consumption, history of allergy for antibioticdiabetics, chronic respiratory disease, hypertension, chronic cardiac dysfunction, cerebrovascular disease, chronic kidney dysfunction, malignant tumor), conditions on bacteremia day (patient department of ICU, length of ICU stay prior to culture, length of hospital stay prior to culture, immunocompromised status, proportion of carbapenem-resistant strains, proportion of multidrug resistant strains, appropriate antimicrobial therapy, antibiotics used prior to culture (n > = 3 types), recent surgery (within 1 mo)), invasive interventions (percutaneous drainage, endotracheal tube, mechanical ventilation, tracheostomy, fiberoptic bronchoscopy, central venous catheter, urinary catheter, gastric tube, special therapy (containing CRRT/ECOMO), enteral nutrition and interventions used prior to culture (n > = 3 types)), laboratory indicators prior to culture (creatinine, blood urea nitrogen (BUN), albumin (ALB), bilirubin, C-reactive protein (CRP), procalcitonin (PCT), lactate (Lac)), disease severity (sequential organ failure assessment score (SOFA), Pitt bacteremia score (PBS) and APACHE II score) and outcomes (28-d mortality, length of hospital stay (day) after onset of bacteremia, survival > 14 d after onset of bacteremia) by thoroughly reviewing medical records. All included patients were divided into pneumonia and nonpneumonia-related AB bacteremia groups. The basic characteristics of the two cohorts are listed in Table 3.

**Table 3:** Demographic and clinical characteristics of 117 patients with pneumonia and non-pneumonia-related *A. baumannii* bacteremia

Patient characteristics	Pneumonia-related bacteremia (*n* = 45)	Nonpneumonia-related bacteremia(*n* = 72)	*P* value
Demographic			
Age (year), median (IQR)	60.664 ± 18.42	60.143 ± 16.57	0.875
Sex, n (%)	34 (75.6%)	50 (69.4%)	0.475
Underlying diseases			
History of allergy to antibiotic, n (%)	6 (13.3%)	6 (8.3%)	0.580
History of smoking, n (%)	27 (60%)	13 (18.1%)	< 0.001
History of alcohol, n (%)	20 (44.4%)	9 (12.5%)	< 0.001
Diabetes, n (%)	13 (28.9%)	14 (19.4%)	0.238
Chronic respiratory disease, n (%)	6 (13.3%)	3 (4.2%)	0.146
Hypertension, n (%)	16 (35.6%)	29 (40.3%)	0.610
Chronic cardiac dysfunction, n (%)	3 (6.7%)	5 (6.9%)	1.000
Chronic kidney dysfunction, n (%)	4 (8.9%)	7 (9.7%)	1.000
Cerebrovascular disease, n (%)	19 (42.2%)	16 (22.2%)	0.022
Malignant tumor, n (%)	7 (15.6%)	22 (30.6%)	0.068
Recent invasive procedures			
Endotracheal tube, n (%)	34 (75.6%)	26 (36.1%)	< 0.001
Mechanical ventilation, n (%)	42 (93.3%)	28 (38.9%)	< 0.001
Tracheostomy, n (%)	22 (48.9%)	9 (12.5%)	< 0.001
Percutaneous drainage, n (%)	23 (51.1%)	46 (63.9%)	0.172
Central venous catheter, n (%)	39 (86.7%)	31 (43%)	< 0.001
Fiberoptic bronchoscopy, n (%)	24 (53.3%)	8 (11.1%)	< 0.001
Indwelling urinary catheter, n (%)	43 (95.6%)	41 (56.9%)	< 0.001
Nasogastric tube, n (%)	43 (95.6%)	32 (44.4%)	< 0.001
Enteral nutrition	24 (53.3%)	37 (51.4%)	0.838
Special therapy (contain CRRT/ECOMO), n (%)	11 (24.4%)	10 (13.9%)	0.148
Invasive interventions used prior to culture (n > = 3 types)	43(95.6%)	35(48.6%)	< 0.001
Conditions on bacteremia day			
Immunocompromised status, n (%)	24 (53.3%)	20 (27.8%)	0.005
Length of hospital stay prior to culture, median (IQR)	12 (8, 23)	7 (5, 11.5)	0.010
Length of ICU stay prior to culture, median (IQR)	10 (2, 18)	0 (0, 6)	< 0.001
Inpatient department of ICU prior to culture, n (%)	40 (88.9%)	31 (43%)	< 0.001
CRAB, n (%)	36 (80%)	33 (45.8%)	< 0.001
MDRAB, n (%)	38 (84.4%)	34 (47.2%)	< 0.001
Antibiotic used (> 3 types) prior to culture, n (%)	31 (68.9%)	18 (25%)	< 0.001
Appropriate antimicrobial therapy, n (%)	17 (37.8%)	38 (52.8%)	0.114
Recent surgery (within 1 mo), n (%)	22 (48.9%)	47 (65.3%)	0.080
Laboratory indicators prior to culture			
Creatinine, median (IQR)	69 (47, 125)	66 (51.5, 103.6)	0.760
Blood urea nitrogen (BUN), median (IQR)	12.85 (7.1, 19.1)	6.74 (5.29, 10.57)	0.001
Albumin (ALB), median (IQR)	15.8 (9.8, 30.6)	20.7 (10.35, 48.5)	0.356
Bilirubin, mean ± sd	31.631 ± 4.5215	33.786 ± 5.4495	0.029
C-reactive protein(CRP), median (IQR)	89.5 (54.57, 153.21)	67.985 (23.055, 127.27)	0.047
Procalcitonin (PCT), median (IQR)	0.741 (0.3, 2.161)	2.833 (0.482, 8.2775)	0.016
Lactate (Lac), median (IQR)	1.3 (1.2, 1.8)	1.6 (1.1, 2.2)	0.137
Disease severity			
SOFA score, median (IQR)	8 (7, 11)	4 (1, 7)	< 0.001
APACHE score, mean ± sd	19.033 ± 5.6598	19.958 ± 6.8872	0.590
PITT score, median (IQR)	6 (4, 9)	2.5 (1, 5)	< 0.001
Outcomes			
28-d mortality, n (%)	35 (77.8%)	31 (43.1%)	< 0.001
Length of hospital stay (d) after onset of bacteremia, n (%)	9 (4.75, 15)	11 (4, 26)	0.510
Survival > 14 d after onset of bacteremia, n (%)	22 (48.9%)	58 (80.6%)	< 0.001

### Definitions

Pneumonia-related AB bacteremia was defined as positive blood and sputum culture with a clinical diagnosis of pneumonia, and nonpneumonia-related AB bacteremia was defined as only positive blood. The diagnosis of pneumonia needed a new or increased infiltration on chest radiography and with at least two of the following signs and symptoms: (1) body temperature greater than 38 °C or lower than 36 °C; (2) heart rate greater than 90 beats per minute; (3) respiratory rate greater than 20 breaths per minute; and (4) a rise in peripheral blood cell count exceeding 10 × 10^9^/L or a fall below 4 × 10^9^/L. HAP was defined as pneumonia that did not exist at the time of hospitalization and was not in the incubation period of infection but occurred 48 h after hospitalization. The onset of BSIs was defined as the day on which the first positive blood culture was collected. Immunosuppression occurred if they had HIV or AIDS, were transplant recipients, had received chemotherapy within the previous 6 weeks, had received systemic therapy for 2 weeks, or had been treated with other immunosuppressive agents within 2 weeks before hospitalization.

### Statistical analysis

Continuous variables were expressed as the mean ± standard deviation or median (interquartile range) and were compared using a two-sample t test or Mann–Whitney U test, depending on whether they were normally distributed. Qualitative variables were expressed as percentages and compared using the chi-square test or Fisher’s exact test. Univariate/multivariate logistic regression analysis was used to explore the independent risk factors in patients with AB bacteremia. Cox regression analysis was used to explore the independent risk factors for mortality in patients with pneumonia-related AB bacteremia, and the results were expressed as odds ratios (ORs) and 95% confidence intervals (CIs). The significance level for statistical testing was defined as two-tailed *p* < 0.05. All statistical analyses were performed using the Statistical Package for the Social Sciences (SPSS, IL, USA), version 25.0, and MedCalc Statistical Software version 18.2.1 (MedCalc Software bv, Ostend, Belgium). Figures were drafted using GraphPad Prism version 8.4.3 (GraphPad Software, CA, USA).

## Results

### Patient characteristics

**Fig. 1:**
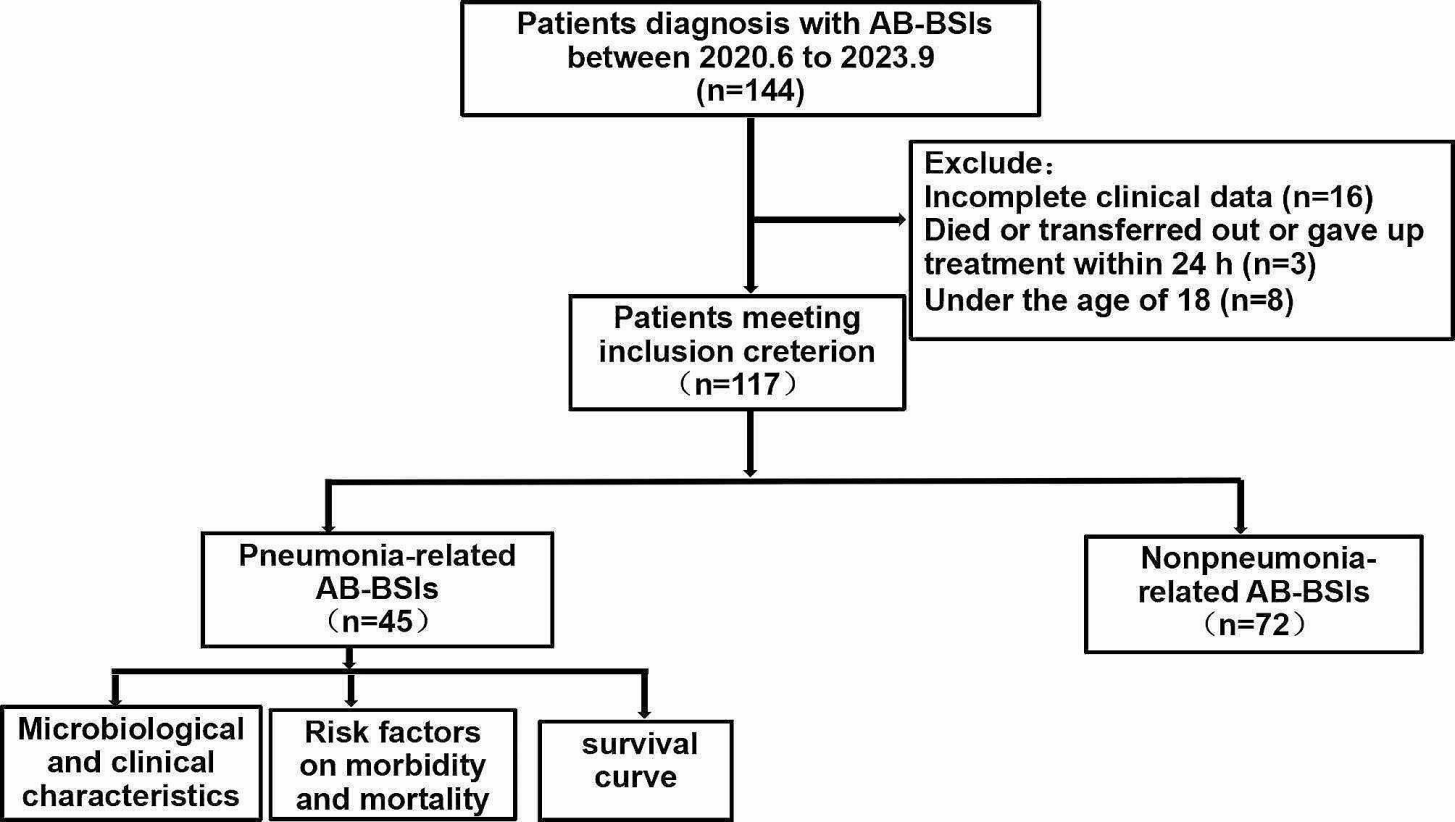
Study flow diagram

A total of 117 patients diagnosed with *A. baumannii* bacteremia were enrolled in this study, among which 84 (71.79%) were males and 33 (28.21%) were females. The pneumonia-related group included 45 patients with a median age of 60.66 ± 18.42 years. The nonpneumonia-related group included 72 patients with a median age of 60.143 ± 16.57 years. The study flow diagram is shown in Fig. 1.

### Drug resistance and sensitivity results in the two groups

Among 117 patients with *A. baumannii* bacteremia, 69 cases (58.97%) were CRAB and 72 cases (61.53%) were MDR. Compared with the nonpneumonia-related bacteremia group, the pneumonia-related bacteremia group had a greater risk of MDRAB infection (84.44%) and CRAB infection (80%) and higher drug resistance rates of compound Xinnuomin (60%), doxycycline (73.17%), cefopetazone/sulbactam (69.76%), ticarcillin/clavulanic acid (85.36%), cefepime (75.5%), meropenem (77.7%), tobramycin (76.74%), minocycline (26.83%), ciprofloxacin (86.05%), piperacillin/tazobactam (83.72%), imipenem (80%), ceftazidime (82.2%), and levofloxacin (77.7%). However, there were no differences in colistin (2.44% vs. 4.17%), tigecycline (6.98% vs. 5.08%), amikacin (40% vs. 11.11%) or compound Xinnuomin (60% vs. 41.6%). From the perspective of drug sensitivity, we found that there was more susceptibility to colistin (97.56% vs. 95.83%) than other antibiotics in the two groups. In the pneumonia-related bacteremia group, only polymyxin had a drug susceptibility rate of over 80%. In contrast to the pneumonia-related bacteremia group, minocycline and amikacin had a relatively high susceptibility rate (> 80% susceptibility) in AB bacteremia patients without pneumonia (Tables 1 and 2).

### The different clinical characteristics of patients with pneumonia-related and nonpneumonia-related AB bacteremia

The 117 patients were divided into two groups with pneumonia (38.46%, 45/117) and nonpneumonia (61.54%, 72/117)-related AB bacteraemia. Table 3 shows their demographics and clinical characteristics. The pneumonia-related group was tended to have the underlying disease of cerebrovascular disease [*P* = 0.022; 19 (42.2%)], inpatient department of ICU prior to culture [*P* < 0.001; 40 (88.9%)], have longer hospital stay (day) prior to culture [*P* = 0.01; 12 (8, 23)], have longer ICU stay (day) prior to culture [*P* < 0.001; 10 (2, 18)], was slightly higher rates of history of smoking [*P* < 0.001; 27 (60%)], history of alcohol [*P* < 0.001; 34 (44.4%)], endotracheal tube [*P* < 0.001; 34 (75.6%)], mechanical ventilation rate [*P* < 0.001; 42 (93.3%)], tracheostomy [*P* < 0.001; 22 (48.9%)], central venous catheter [*P* < 0.001; 39 (86.7%)], fiberoptic bronchoscopy [*P* < 0.001; 24 (53.3%)], indwelling urinary catheter [*P* < 0.001; 43 (95.6%)], nasogastric tube [*P* < 0.001; 43 (95.6%)], invasive interventions used prior to culture (n > = 3 types) [*P* = 0; 43 (95.6%)], immunocompromised status [*P* = 0.005; 24 (53.3%)], CRAB [*P* < 0.001; 36 (80%)], MDRAB [*P* < 0.001; 38 (84.4%)], antibiotic used (> 3 types) prior to culture [*P* < 0.001; 31 (68.9%)], 28-d mortality [*P* < 0.001; 35 (77.8%)] and survival > 14 d after onset of bacteremia [*P* < 0.001; 22 (48.9%)]. Compared with the nonpneumonia-related bacteremia group, the pneumonia-related bacteremia group had a high level of blood urea nitrogen [*P* = 0.001; 12.85 (7.1, 19.1)], C-reactive protein [*P* = 0.047; 89.5 (54.57, 153.21)], SOFA score [*P* < 0.001; 8((7, 11)], and PITT score [*P* < 0.001; 6 (4, 9)]. However, the patients with pneumonia had lower levels of procalcitonin (*P* = 0.016) and bilirubin (*P* = 0.029). There were no significant differences in APACHE II score, lactate, albumin, creatinine, appropriate antimicrobial therapy, special therapy (containing CRRT/ECOMO), percutaneous drainage, recent surgery, underlying disease of malignant tumor, chronic kidney dysfunction, chronic cardiac dysfunction, hypertension, chronic respiratory disease, diabetes, enteral nutrition and history of allergy to antibiotics.

### Risk factors for incidence of pneumonia-related AB bacteremia

Univariate analysis showed that survival time (day), CRAB, MDRAB, length of hospital stay prior to culture, length of ICU stay prior to culture, immunocompromised status, antibiotics used prior to culture (n > = 3 types), endotracheal tube, fiberoptic bronchoscopy, PITT, SOFA and invasive interventions used prior to culture (n > = 3 types) were associated with pneumonia-related AB bacteremia. After adjusting for confounding factors, the results of multivariate logistic regression analysis revealed that recent surgery (within 1 mo) [*P* = 0.043; 0.306 (0.098–0.962)] and invasive interventions (n > = 3 types) [*P* = 0.021; 0.072 (0.008–0.671)] were independent risk factors related to pneumonia-related AB bacteremia (Table 4).

**Table 4:** Risk factors for the incidence of pneumonia-related *A. baumannii* bacteremia

Characteristics	Total (*n*)	Univariate analysis			Multivariate analysis	
		Odds Ratio (95% CI)	*P* value		Odds Ratio (95% CI)	*P* value
Sex	84	1.360 (0.584–3.165)	0.476			
Age	117	1.002 (0.980–1.024)	0.873			
Survival time(d)	117	1.035 (1.010–1.061)	**0.006**		1.027 (0.987–1.069)	0.186
CRAB	48	0.212 (0.089–0.502)	**< 0.001**		2.442 (0.048–123.012)	0.655
MDRAB	45	0.165 (0.065–0.418)	**< 0.001**		0.255 (0.005–13.487)	0.500
Length of hospital stay prior to culture (d)	117	1.041 (1.006–1.077)	**0.023**		0.982 (0.929–1.039)	0.530
Length of ICU stay prior to culture (d)	117	1.119 (1.057–1.186)	**< 0.001**		1.001 (0.924–1.084)	0.982
Recent surgery (within 1 mo)	69	0.509 (0.238–1.088)	0.081		0.306 (0.098–0.962)	**0.043**
Immunocompromised status	44	2.914 (1.334–6.366)	**0.007**		1.678 (0.577–4.880)	0.342
Antibiotics used prior to culture (n > = 3 types)	49	6.643 (2.907–15.179)	**< 0.001**		1.210 (0.368–3.982)	0.754
Endotracheal tube	57	0.183 (0.080–0.421)	**< 0.001**		1.241 (0.318–4.839)	0.755
Fiberoptic bronchoscopy	32	9.143 (3.572–23.400)	**< 0.001**		3.103 (0.903–10.660)	0.072
PITT	117	1.313 (1.158–1.489)	**< 0.001**		1.013 (0.728–1.408)	0.940
SOFA	117	1.265 (1.136–1.408)	**< 0.001**		1.009 (0.756–1.347)	0.952
Invasive interventions (n > = 3 types)	39	0.044 (0.010–0.195)	**< 0.001**		0.072 (0.008–0.671)	**0.021**

### Risk factors for mortality in patients with pneumonia-related AB bacteremia

**Fig. 2:**
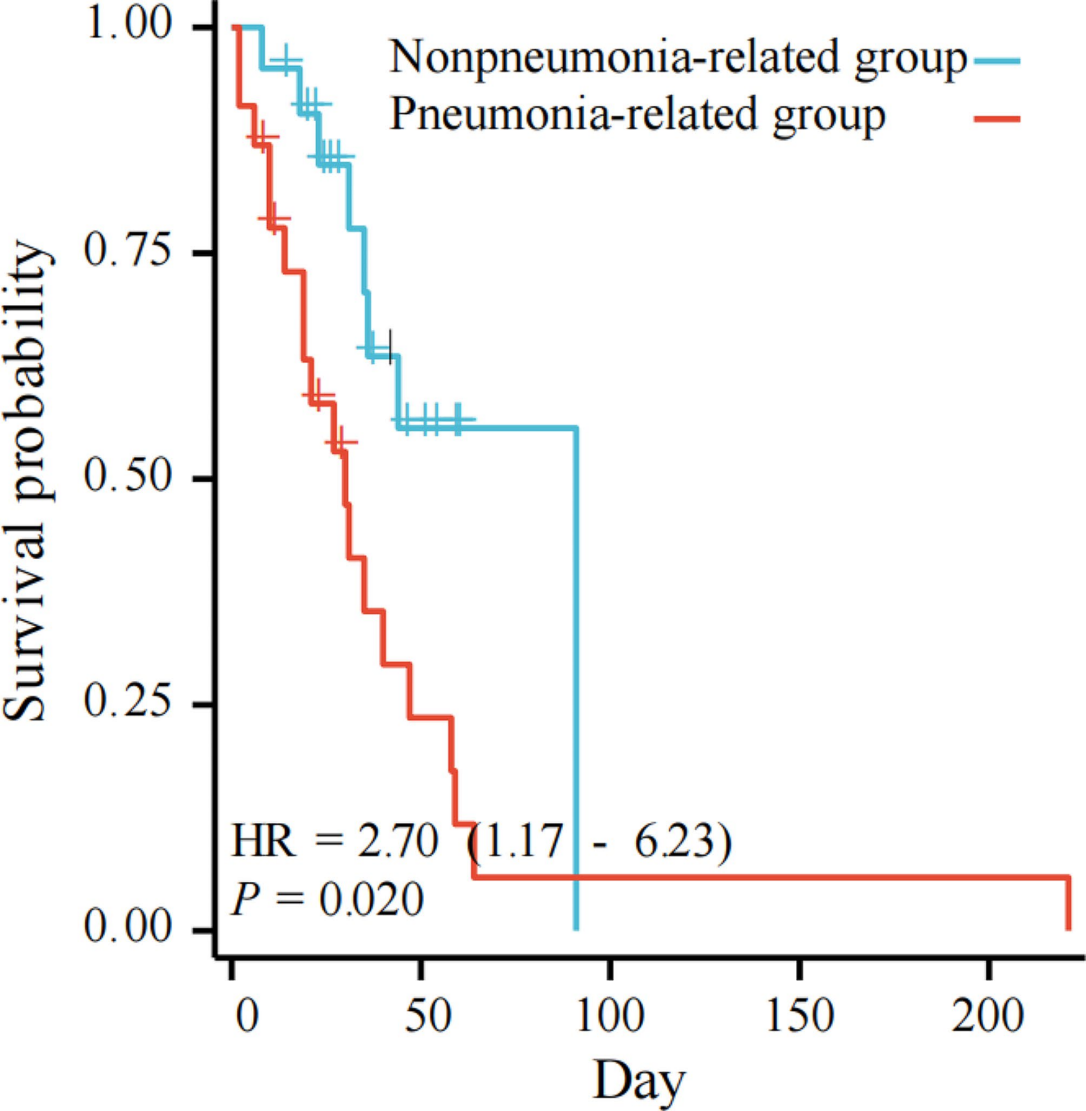
The Kaplan‒Meier curve and the timing test of the two groups

As shown in Table 5, the length of ICU stay prior to culture and recent surgery (within 1 mo) were associated with the mortality and survival time of patients with pneumonia-related AB bacteremia. After adjusting for confounding factors, multivariate logistic regression analysis revealed that the length of ICU stay prior to culture [*P* = 0.009; 0.959 (0.930–0.990)] and recent surgery [*P* = 0.004; 0.260 (0.105–0.646)] were also independent risk factors for mortality in patients with pneumonia-related AB bacteremia. The Kaplan‒Meier curve and the timing test showed that recent surgery [*P* = 0.02; 2.70 (1.17–6.23)] was associated with the survival time of patients with pneumonia-related AB bacteremia (Fig. 2).

**Table 5:** Risk factors for mortality of the patients with pneumonia-related *A. baumannii* bacteremia

Characteristics	Total (*n*)	Univariate analysis			Multivariate analysis	
		Hazard ratio (95% CI)	*P* value		Hazard ratio (95% CI)	*P* value
Sex	34	0.953 (0.407–2.231)	0.912			
Age	45	1.002 (0.980–1.024)	0.877			
CRAB	9	0.877 (0.349–2.206)	0.780			
Length of ICU stay prior to culture (d)	45	0.970 (0.941–1.000)	**0.049**		0.959 (0.930–0.990)	**0.009**
Recent surgery (within 1 mo)	22	0.371 (0.160–0.857)	**0.020**		0.260 (0.105–0.646)	**0.004**
Immunocompromised status	24	0.786 (0.362–1.706)	0.543			
Endotracheal tube	11	1.120 (0.443–2.832)	0.811			
Fiberoptic bronchoscopy	24	0.614 (0.273–1.384)	0.239			
PITT	45	1.132 (0.997–1.286)	0.055		1.063 (0.949–1.191)	0.292
SOFA	45	1.074 (0.939–1.228)	0.298			
Invasive interventions (n > = 3 types)	2	0.783 (0.104–5.862)	0.811			

## Discussion

Bacteremia refers to the systemic infection caused by pathogenic microorganisms entering the bloodstream, often leading to sepsis and septic shock. In severe cases, it can cause multiple organ failure and even death. According to the Infectious Diseases Society of America (IDSA) 2023 Guidance, Carbapenem-resistant A. baumannii (CRAB) infections pose significant challenges in healthcare settings because of their uncertain antibiotic therapy and complex host status [16]. The mortality of paitients with A. baumannii bacteremia is gradually increasing due to its widespread resistance [17]. Some studies have shown that patients with pneumonia-related A. bau*mannii* (AB) bacteremia have poorer drug sensitivity and prognosis than those with nonpneumonia-related AB bacteremia [14, 18]. Therefore, we mainly analyzed the characteristics of antibiotic resistance and clinical characteristics and explored the main risk factors for incidence and prognosis in patients with pneumonia-related AB bacteremia.

As shown in Table 1, the resistance rates to imipenem, meropenem, tigecycline and colistin were 58.9%, 78.89%, 5.88% and 3.37%, respectively, which is similar to the data in the CHINET data [19]. We also found that the pneumonia-related bacteremia group had a high resistance rate of CRAB to 80%, limiting the clinical treatment options. Studies have shown that an over 25% reduction in the mortality rate for AB bacteremia is associated with the early initiation of adequate empirical antimicrobial therapy [20, 21]. So we also analysed in vitro susceptibility results of pneumonia-related AB bacteremia to major drugs. As shown in Table 2, only colistin had > 80% susceptibility in vitro in patients with pneumonia-related AB bacteremia. For moderate to severe infection caused by CRAB, the IDSA panel suggests combination therapy, preferably with two agents demonstrating in vitro activity [16]. And for bloodstream infections, ampicillin-sulbactam with cefiderocol or polymyxin B is preferred [22]. It is also supported by Kim et al. and Yu et al., who revealed that early colistin therapy was an independent favorable prognostic factor associated with 28-day mortality in patients with CRAB bacteremia [23]. Polymyxin may be considered to be used for the treatment of pneumonia-related bacteremia infections.

Our research revealed that some clinical characteristics were significantly different between patients with pneumonia AB and nonpneumonia AB bacteremia. A Univariate analysis revealed that pneumonia-related group tended to had higher rates of MDRAB and CRAB. This result may be due to patients with the pneumonia-related AB bacteremias had more distinct hospital and antibiotics exposure [24], which also was confirmed by our research. We also found that the pneumonia-related group had more rate of invasive procedures, the higher PITT scores, the higher SOFA scores, and the poorer outcome.

Furthermore, a multivariate analysis revealed that recent surgery (within 1 mo) and invasive interventions were independent risk factors for the acquisition of pneumonia-related AB bacteraemia. As we known, A. baumannii has been found to have the ability to form biofilms, which is a effective way for the bacteria not only to survive in the presence of antibiotics, but also survive for long periods on the surfaces of medical devices [25]. And bacteremia may develop after the disruption of the skin and mucosal barrier of patients through invasive procedures and some surgeries [26]. These all provided conditions for the occurrence of bacteremia in patients.

The other multivariate analysis revealed that the length of ICU stay prior to culture and recent surgery were independent risk factors for mortality of pneumonia-related AB bacteremia. Patients in the ICU are typically in critical conditions, have low immune function, and undergo invasive procedures [27]. And patients who are long stay in the ICU indicates that their conditions were more severe, directly leading to a poor prognosis. Patients who undergo certain surgery often have been associated with increased hospital length of stays, the ICU length of stays, and morbidity resulting from pneumonia [28]. And bacteremia often occurrs more after surgery [26]. Considering the severe contions, long-term hospitalization and immunocompromised status, patients who undergo recent surgery often have a poor prognosis [29]. We also confirmed that the survival time of pneumonia-related AB bacteremia group was significantly shorter than that of nonpneumonia-related AB bacteremia group by Kaplan‒Meier curve.

This study has the following limitations: (1) without involving antibiotic treatments, including antibiotic exposure, empiric antibiotic therapy, appropriate empiric antibiotic therapy, definite antibiotic therapy and the new antibiotics, which may affect the primary outcome; (2) since the included cases were single-center samples, the results cannot be generalized to other regions; (3) this study is prone to bias because of its retrospective characteristics; and (4) because of the small sample size, the research results and conclusions are only for reference. We suggest that future studies from different institutes and different geographic areas evaluate the efficacy of recent surgery in predicting mortality from *A. baumannii* bacteremia.

In summary, patients with pneumonia-related AB bacteremia had poorer antibiotic susceptibility and outcome, and only polymyxin had a drug susceptibility rate of over 80% in vitro. Recent surgery(within 1 mo) was a significantly independent predictor in patients with pneumonia-related AB bacteremia. Therefore, we should pay more attention to the management of surgical patients. The hospital should strengthen the monitoring of environmental hygiene and the prevention and control policies of nosocomial infections, improve the compliance of the medical staff and inpatient wards with regular hand-hygiene practices.

### Electronic supplementary material

Below is the link to the electronic supplementary material.

Supplementary Material 1

## Data Availability

The datasets used and/or analyzed during the current study are available from the corresponding author upon reasonable request.

## Abbreviations

AB: *Acinetobacter baumannii*
AB: BSIs *Acinetobacter baumannii* bloodstream infections
CRAB: Carbapenem-resistant *A. baumannii*
MDRAB: Multidrug resistant *A. baumannii*
ICU: Intensive care unit
