# Supplementary material for: Risk factors for bacteremic pneumonia and mortality (28-day mortality) in patients with Acinetobacter baumannii bacteremia
Source: BMC Infect Dis. 2024 Apr 26;24:448. doi: 10.1186/s12879-024-09335-8 (PMC11046916; doi:10.1186/s12879-024-09335-8)
Supplement: Supplementary file 1 — Supplementary Material 1 [file 12879_2024_9335_MOESM1_ESM.docx]

**Supplementary table 1 The details type of surgeries between pneumonia and non-pneumonia-related *A. baumannii* bacteremia**

| [Region](file:///C:/Users/liangjing/AppData/Local/Programs/baidu-translate-client/resources/app.asar/app.html#/#) of surgeries | Pneumonia-related  bacteremia (n=22) | Nonpneumonia-related bacteremia(n=47) | P value |
| --- | --- | --- | --- |
| Head and face, n (%) | 10 (45.5%) | 11 (23.4%) | 0.064 |
| Chest, n (%) | 3 (13.6%) | 2 (4.3%) | 0.367 |
| Abdomen, n (%) | 7 (31.8%) | 23 (48.9%) | 0.181 |
| Limbs and soft tissues, n (%) | 1 (4.5%) | 4 (8.5%) | 0.925 |
| Urinary system, n (%) | 1 (4.5%) | 2 (4.3%) | 1 |
| Others, n (%) | 1 (4.5%) | 0 (0) |  |
